# Real-world antibiotic use in treating acute exacerbations of chronic obstructive pulmonary disease (AECOPD) in China: Evidence from the ACURE study

**DOI:** 10.3389/fphar.2021.649884

**Published:** 2021-05-25

**Authors:** Yiming Ma, Ke Huang, Chen Liang, Xihua Mao, Yaowen Zhang, Zijie Zhan, Ting Yang, Yan Chen

**Affiliations:** ^1^ Department of Pulmonary and Critical Care Medicine, The Second Xiangya Hospital, Central South University, Changsha, China; ^2^ Department of Pulmonary and Critical Care Medicine, China–Japan Friendship Hospital, Beijing, China; ^3^ National Clinical Research Center for Respiratory Diseases, Beijing, China; ^4^ Institute of Respiratory Medicine, Chinese Academy of Medical Science, Beijing, China; ^5^ Chinese Alliance for Respiratory Diseases in Primary Care, Beijing, China

**Keywords:** chronic obstructive pulmonary disease, exacerbation, antibiotic, bacterial infection, treatment

## Abstract

**Background:** The evidence for real-world antibiotic use in treating acute exacerbations of chronic obstructive pulmonary disease (AECOPD) is insufficient. This study aimed to investigate real-world antibiotic use in the management of AECOPD in China.

**Methods:** All hospitalized AECOPD patients from the acute exacerbation of chronic obstructive pulmonary disease inpatient registry (ACURE) study conducted at 163 sites between January 2018 and December 2019 were screened according to the eligible criteria. The eligible study population was divided into secondary and tertiary hospital groups. Patients’ baseline characteristics, antibiotic use, and bacterial pathogen characteristics were retrieved and analyzed using SPSS 23.0.

**Results:** A total of 1663 patients were included in the study, including 194 patients from secondary hospitals and 1469 patients from tertiary hospitals. Among the 1663 AECOPD patients enrolled, 1434 (86.2%) received antibiotic treatment, comprising approximately 85.6% and 86.3% of patients in the secondary and tertiary hospital groups, respectively. The median antibiotic therapy duration was 9.0 (interquartile range [IQR]: 7.0 - 11.0)°days. Regarding the routes of antibiotic use, 1400 (97.6%) patients received intravenous antibiotics, 18 (1.3%) patients received oral antibiotics, 15 (1.0%) patients received both intravenous and oral antibiotics, and one (0.1%) patient received both oral and nebulized antibiotic treatment. In addition, cephalosporin, penicillin, and quinolone were the most commonly prescribed antibiotics (43.6%, 37.0%, and 34.2%, respectively). In total, 990 (56.5%) patients underwent pathogen examinations; the proportion of patients receiving pathogen examinations in the second hospital group was significantly lower than that in the tertiary hospital group (46.4% vs 61.3%, p < 0.001).

**Conclusion:** This study demonstrates that an antibiotic overuse may exist in the treatment of AECOPD in China. Measures should be taken to prevent the overuse of antibiotics and potential antimicrobial resistance (AMR) in Chinese AECOPD patients.

## Introduction

According to the Global Initiative for Chronic Obstructive Lung Disease (GOLD) 2021 report, (Global Strategy for the Diagnosis, Management, 2020) chronic obstructive pulmonary disease (COPD) is a common, preventable, and treatable disease, characterized by persistent respiratory symptoms and airflow limitation due to airway and/or alveolar abnormalities that are usually caused by significant exposure to noxious particles or gases and influenced by host factors, including abnormal lung development. In China, the prevalence of COPD among individuals aged 40°years and older is 13.7%, and cigarette smoking, ambient air pollution, underweight, childhood chronic cough, parental history of respiratory diseases, and low education are major risk factors for COPD. (Wang et al., 2018) Additionally, COPD was the fifth leading cause of death in China in a survey during 1980–2013, (Global, Regional, and National Age-Sex Specific Mortality for 264 Causes of Death, 2017) and is now the third leading cause of death all over the world, (Naghavi et al., 2015) indicating that COPD has become a significant public health problem.

Acute exacerbations of chronic obstructive pulmonary disease (AECOPD) are characterized by episodes of worsening of symptoms, resulting in substantial morbidity and mortality. (Wedzicha and Seemungal, 2007) Per the GOLD 2021 report, AECOPD can be classified as mild, moderate, and severe, based on the severity of the disease. (Global, 2020) Patients with mild AECOPD should only be treated with short-acting bronchodilators (SABDs), those with moderate AECOPD are treated with SABDs plus antibiotics and/or oral corticosteroids, while those with severe AECOPD require hospitalization or visits to the emergency room. As there is heterogeneity in the biological response, COPD exacerbations can be divided into different phenotypes, such as bacteria-, virus-, or eosinophil-associated exacerbations. (Bafadhel et al., 2011) Specifically, respiratory bacterial infection is a major risk factor for the pathogenesis of COPD. (Beasley et al., 2012) Bacterial infections during AECOPD are common; a recent meta-analysis involving 118 studies between 1980 and 2018 has revealed that the overall estimation of the prevalence of bacterial infection in AECOPD is 49.59%. (Moghoofei et al., 2020)

Prompt treatment improves recovery from exacerbation, reduces the risk of hospitalization, and is associated with a better health-related life quality of AECOPD patients. (Wilkinson et al., 2004) Previous studies have suggested that antibiotic use might influence the prognosis of AECOPD, including treatment failure and rehospitalization. (Ruiz-gonzález et al., 2007; Vollenweider et al., 2018) The GOLD 2021 report suggested that antibiotic choice should be based on the local bacterial resistance pattern, and the initial empirical treatment is usually an aminopenicillin with clavulanic acid, macrolide, or tetracycline; importantly, the report emphasized the potential likelihood of gram-negative bacterial infection (e.g., *Pseudomonas* species). (Global, 2020) National Institute for Health and Clinical Excellence (NICE) guideline from the United Kingdom for AECOPD management provides a list of the first-choice oral antibiotics (including amoxicillin, doxycycline, and clarithromycin) and first-choice intravenous antibiotic (including amoxicillin, co-amoxiclav, clarithromycin, co-trimoxazole, and piperacillin with tazobactam); notably, the NICE guideline stresses that clinicians should assess the effects of intravenous antibiotics within 48 hours and consider stepping down to oral antibiotics when possible. (NICE guideline [NG114], 2020) Moreover, the GOLD 2021 report indicates that antibiotics are among the three most commonly used classes of medications for treating COPD exacerbations; antibiotics should be administered to AECOPD patients with clinical signs of bacterial infection, for a recommended duration of 5-7°days. (Global, 2020) However, the evidence for antibiotic use in general clinical practice when treating AECOPD is insufficient.

Hospitals in China are classified into grades I, II, and III based on their functions and roles: Grade I hospitals include community health centers and township health centers directly providing preventive care, medical care, and rehabilitation services for residents; Grade II hospitals are secondary hospitals providing comprehensive medical services to a region and conducting limited teaching and scientific research; and Grade III hospitals are tertiary hospitals providing high-level specialized medical services and conducting advanced teaching and scientific research.(Li et al., 2020) Significant variations in medical care quality have been observed among these three grades of hospitals. (Xu et al., 2020) Furthermore, previous studies have demonstrated a relationship between hospital-grade quality of care and patients’ clinical outcomes. (Nakano et al., 2019; He et al., 2021) This study aimed to investigate real-world antibiotic use in AECOPD patients in Chinese hospitals of different grades.

## Methods

### Study design

All data were obtained from the database of the acute exacerbation of chronic obstructive pulmonary disease inpatient registry (ACURE) study (ClinicalTrials.gov registry number: NCT02657525). The ACURE study was an ongoing, national, multicenter, observational registry aimed to investigate the overall clinical features and treatment procedures of hospitalized Chinese AECOPD patients in a real-world setting. The enrollment of participants in the ACURE study formally began in September 1, 2017, and the expected end of patient enrollment in all centers was December 2019. The ACURE study planned to recruit 7600 in-hospital AECOPD patients with a 3-year follow-up. By December 2019, 163 secondary and tertiary sites were finally included in the ACURE study, and all sites were distributed in 28 provinces across China. The details of the ACURE study design have been published elsewhere. (Pei et al., 2020) The study was approved by the Ethics Committee for China-Japan Friendship Hospital (2015-88). Informed consent was obtained from all involved patients. This study was conducted in accordance with the ethical standards formulated in the Helsinki Declaration.

### Study participants

Data of all hospitalized AECOPD patients from all 163 sites in the ACURE study conducted between January 2018 and December 2019 were reviewed. The eligible criteria for AECOPD patients were as follows: 1) age no less than 18°years old; 2) who agreed to sign an informed consent for their participation in the ACURE study; 3) who did not participate in other clinical trials or intervention studies; 4) who had a confirmed diagnosis of AECOPD at discharge per the GOLD 2017 report; 5) with spirometry-verified COPD, which was defined as post-bronchodilator forced expiratory volume in one second (FEV_1_)/forced vital capacity (FVC) <0.70; 6) who had no primary diagnosis of other respiratory diseases related to hospitalization other than AECOPD (including asthma, bronchiectasis, pulmonary tuberculosis, pneumonia, pulmonary interstitial fibrosis, lung cancer, pulmonary artery hypertension, and pulmonary embolism, etc.). The eligible study population was divided into a secondary and a tertiary hospital group based on the grade of admitted hospitals.

### Data sources and study outcomes

For each patient, we extracted age, gender, body mass index (BMI), smoking status, comorbidities, hospitalization frequency due to AECOPD in the past year, COPD assessment test (CAT) score at admission and post-bronchodilator FEV_1_/FVC. Data of antibiotic use and bacterial pathogen characteristics during admission were also reviewed. The primary outcomes were antibiotic using characteristics and antibiotic prescriptions for AECOPD patients. The secondary outcomes were the bacterial pathogen characteristics of AECOPD patients.

### Statistical methods

The median and interquartile range (IQR) were used to describe continuous variables with a skewed distribution, while continuous variables with a normal distribution were presented as the mean and standard deviation (SD). Categorical variables were expressed as frequencies and percentages. The Mann-Whitney U test was performed to compare continuous variables with a skewed distribution, whereas the independent sample t test was conducted to compare continuous variables with a normal distribution. The chi-square test or Fisher’s exact test was used to compare categorical variables.

A two-side p-value of less than 0.05 was defined as statistically significant. SPSS Windows Version 23.0 (IBM Corporation, Armonk, NY, USA) was used to perform statistical analysis.

## Results

### Baseline characteristics of included AECOPD patients

**FIGURE 1:**
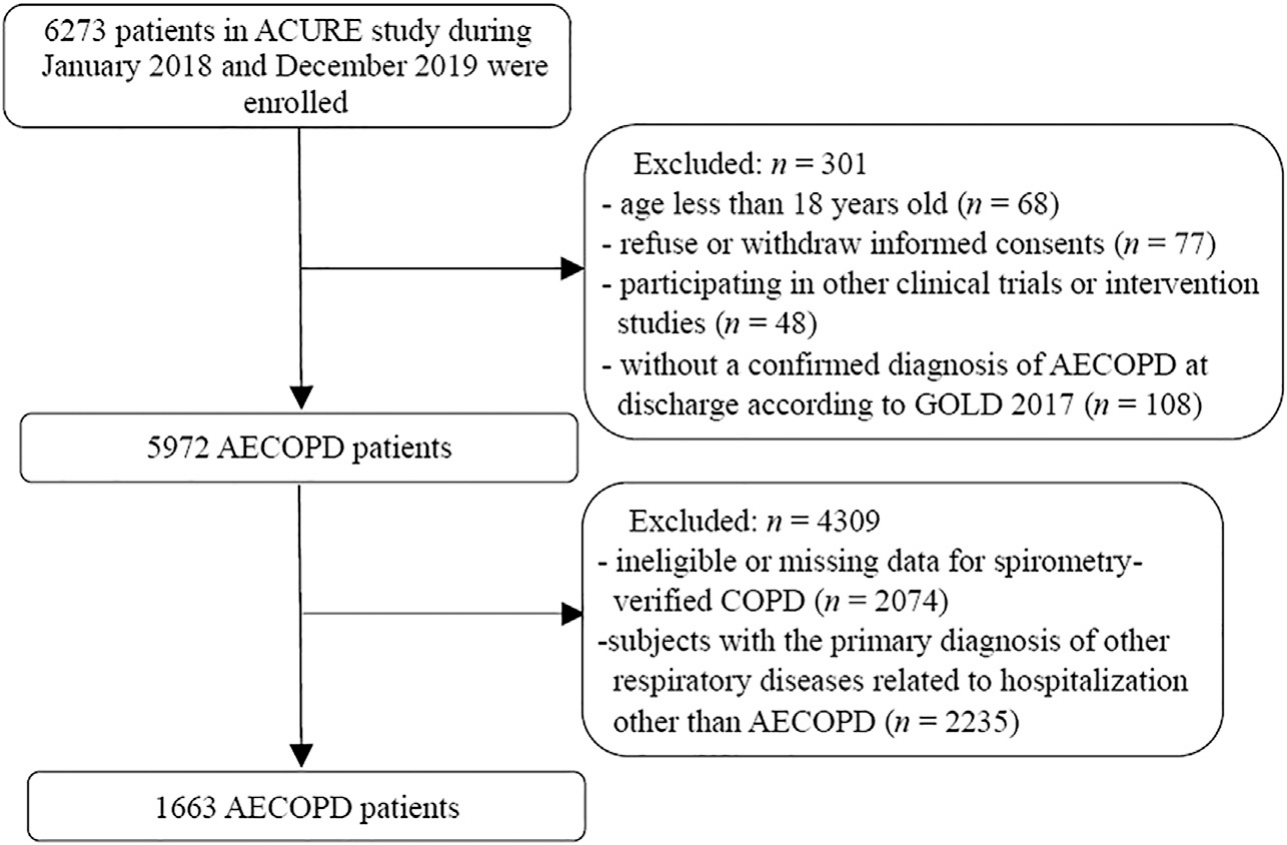
Flowchart diagram of eligible study population.

Finally, a total of 1663 patients who met the eligibility criteria were included in the study (Figure 1). In the study population, 194 and 1469 patients were admitted in the secondary and tertiary hospitals, respectively. The baseline characteristics of included AECOPD patients are demonstrated in Table 1. Of the total study population, 1318 (79.3%) were men and 345 (20.7%) were women. The median age of the overall study population was 70.0 (IQR: 64.0 - 77.0)°years, and the median BMI was 22.1 (IQR: 19.8 - 24.5) kg/m^2^. A total of 1122 (67.5%) patients had at least one comorbidity other than COPD. Of the 1663 included patients, the median hospitalization frequency due to AECOPD in the past year was 0.0 (IQR: 0.0 - 1.0). The median of CAT scores at admission and post-bronchodilator FEV_1_/FVC were 19.0 (IQR: 15.0 - 24.0) and 0.50 (IQR: 0.42 - 0.59), respectively. There was a markedly higher CAT score at admission in the secondary hospital group than the tertiary hospital group (p < 0.001).

**TABLE 1:** Baseline characteristics of included AECOPD patients.

Characteristics	Overall (*n*=1663)	Secondary hospital (*n*=194)	Tertiary hospital (*n*=1469)	p - value
**Age, years**	70.0 (64.0 - 77.0)	71.5 (65.8 - 77.0)	70.0 (64.0 - 77.0)	0.149
**Gender, male**	1318 (79.3)	160 (82.5)	1158 (78.8)	0.239
**BMI, kg/m** ^**2**^	22.1 (19.8 - 24.5)	22.4 (20.1 – 25.0)	22.0 (19.8 - 24.4)	0.358
**Smoking status**				0.750
**Current smoker**	480 (28.9)	54 (27.8)	426 (29.0)	
**Ex-smoker**	704 (42.3)	87 (44.8)	617 (42.0)	
**Never smoking**	479 (28.8)	53 (27.3)	426 (29.0)	
**Any comorbidity**	1122 (67.5)	127 (65.5)	995 (67.7)	0.526
**Cardiovascular diseases**	748 (50.5)	98 (50.5)	650 (44.2)	0.099
**Endocrine and metabolic diseases**	167 (10.0)	16 (8.2)	151 (10.3)	0.376
**Digestive diseases**	57 (3.4)	4 (2.1)	53 (3.6)	0.266
**Other diseases**	645 (38.8)	67 (34.5)	578 (39.3)	0.196
**Hospitalization frequency due to AECOPD in the past year, times**	0 (0 - 1.0)	0 (0 - 2.0)	0 (0 - 1.0)	0.238
**CAT score at admission**	19.0 (15.0 - 24.0)	22.0 (15.0 - 29.0)	19.0 (15.0 - 24.0)	< 0.001
**Post-bronchodilator FEV**_**1**_ **/FVC**	0.50 (0.42 - 0.59)	0.50 (0.40 - 0.59)	0.50 (0.43 - 0.59)	0.285

Notes: Values are presented as median (IQR) or number (percentage).

Legend: AECOPD – acute exacerbation of chronic obstructive pulmonary disease; BMI - body mass index; CAT - COPD Assessment Test; FEV_1_ - forced expiratory volume in one second; FVC: forced vital capacity.

### Antibiotic use characteristics of included AECOPD patients receiving antibiotic treatment

Among the 1663 AECOPD patients enrolled, 1434 (86.2%) received antibiotic treatment. Approximately 85.6% and 86.3% of patients in the secondary and tertiary hospital groups, respectively, received such treatment. Overall, 1102 (76.8%) patients were treated with monotherapy, while 332 (23.2%) received combination therapy with ≥2 antibiotics. In addition, the proportion of patients in the secondary hospital group receiving combination therapy with ≥2 antibiotics was significantly lower than that in the tertiary hospital group (7.8% vs 25.2%, p < 0.001). As for the duration of antibiotic use, the median time in the overall population was 9.0 (IQR: 7.0 - 11.0)°days. Compared with the secondary hospital group, the tertiary hospital group demonstrated a significantly longer median duration of therapy (9.0 vs. 8.0°days, *p*<0.05). With regard to the routes of antibiotic use, 1400 (97.6%) patients received intravenous antibiotics, 18 (1.3%) received oral antibiotics, 15 (1.0%) received both intravenous and oral antibiotics, and one (0.1%) patient received both oral and nebulized antibiotic treatment (Table 2).

**TABLE 2:** Antibiotic using characteristics of included AECOPD patients receiving antibiotic treatment.

Characteristics	Overall (*n*=1434)	Secondary hospital (*n*=166)	Tertiary hospital (*n*=1268)	p - value
**Types of antibiotic use**				< 0.001
**Monotherapy**	1102 (76.8)	153 (92.2)	949 (74.8)	
**Combination therapy with ≥2 antibiotics**	332 (23.2)	13 (7.8)	319 (25.2)	
**Duration of antibiotic use, days (*n*=1400)**	9.0 (7.0 – 11.0)	8.0 (6.0 – 11.0)	9.0 (7.0 – 11.0)	0.030
**Routes of antibiotic use**				0.242
**Oral**	18 (1.3)	0 (0)	18 (1.4)	
**Intravenous**	1400 (97.6)	166 (100)	1234 (97.3)	
**Oral + intravenous**	15 (1.0)	0 (0)	15 (1.2)	
**Oral + nebulized**	1 (0.1)	0 (0)	1 (0.1)	

Notes: Values are presented as median (IQR) or number (percentage).

Legend: AECOPD – acute exacerbation of chronic obstructive pulmonary disease.

### Antibiotic prescriptions of included AECOPD patients receiving antibiotic treatments

Table 3 shows antibiotic prescriptions of the included AECOPD patients who received antibiotic treatment. Of 1434 AECOPD patients receiving antibiotic treatment, the proportions of cephalosporin, penicillin, and quinolone use were 43.6%, 37.0% and 34.2%, respectively. In addition, the proportions of anti-*Pseudomonas* cephalosporin, penicillin and anti-*Pseudomonas* penicillin in the tertiary hospital group were significantly higher than the secondary hospital group (all p < 0.01). Moreover, quinolone and anti-*Pseudomonas* quinolone were more commonly prescribed in secondary hospitals in contrast with tertiary hospitals (both p < 0.01).

**TABLE 3:** Antibiotic prescriptions of included AECOPD patients receiving antibiotic treatment.

Characteristics	Overall (*n*=1434)	Secondary hospital (*n*=166)	Tertiary hospital (*n*=1268)	p - value
**Cephalosporin**	625 (43.6)	72 (43.4)	553 (43.6)	0.954
**Anti-*Pseudomona*s cephalosporin**	281 (19.6)	19 (11.4)	262 (20.7)	0.005
**Penicillin**	530 (37.0)	30 (18.1)	500 (39.4)	< 0.001
**Anti-*Pseudomonas* penicillin**	465 (32.4)	21 (12.7)	444 (35.0)	< 0.001
**Quinolone**	491 (34.2)	76 (45.8)	415 (32.7)	
**Anti-*Pseudomonas* quinolone**	329 (22.9)	56 (33.7)	273 (21.5)	0.001
**Aminoglycoside**	48 (3.3)	0 (0)	48 (3.8)	0.001
**Macrolide**	33 (2.3)	0 (0)	33 (2.6)	0.011
**Carbapenem**	30 (2.1)	1 (0.6)	29 (2.3)	0.068
**Aztreonam**	5 (0.3)	0 (0)	5 (0.4)	0.255
**Clindamycin**	4 (0.3)	0 (0)	4 (0.3)	> 0.999

Notes: Values are presented as median (IQR) or number (percentage).

Legend: AECOPD – acute exacerbation of chronic obstructive pulmonary disease.

### Bacterial pathogen characteristics of included AECOPD patients receiving pathogen examinations

Among 1663 included AECOPD patients, 990 (56.5%) underwent at least one pathogen examination. Notably, the proportion of patients receiving pathogen examinations in the second hospital group was significantly lower than that in the tertiary hospital group (46.4% vs 61.3%, p < 0.001). A qualified sputum was the main specimen source of pathogen examination, followed by blood, nasopharyngeal swab, alveolar lavage fluid, and pleural effusion. The proportion of qualified sputum specimen sources in the tertiary hospital group was significantly higher than that in the secondary hospital group (96.2% vs. 65.6%, p < 0.001), whereas the proportion of nasopharyngeal swab specimen sources was markedly lower than that in the secondary hospital group (1.6% vs.34.4%, p < 0.001) (Table 4). A total of 775 AECOPD patients had bacterial culture results during admission. The most common bacterial pathogens isolated were *Pneumonia klebsiella*, *Acinetobacter*, *Haemophilus influenzae*, *Pseudomonas aeruginosa,* and *Streptococcus pneumoniae* isolates (Table 5).

**TABLE 4:** Specimen sources of included AECOPD patients receiving pathogen examinations.

Characteristics	Overall (*n*=990)	Secondary hospital (*n*=90)	Tertiary hospital (*n*=900)	p - value
**Qualified sputum**	925 (93.4)	59 (65.6)	866 (96.2)	< 0.001
**Nasopharyngeal swabs**	45 (4.5)	31 (34.4)	14 (1.6)	< 0.001
**Blood**	53 (5.4)	7 (7.8)	46 (5.1)	0.409
**Alveolar lavage fluid**	13 (1.3)	0 (0)	13 (1.4)	0.508
**Pleural effusion**	1 (0.1)	0 (0)	1 (0.1)	> 0.999

Notes: Values are presented as number (percentage).

Legend: AECOPD – acute exacerbation of chronic obstructive pulmonary disease.

**TABLE 5:** Distribution of positive bacterial pathogens in included AECOPD patients with bacterial culture results.

Characteristics	Overall (*n*=775)	Secondary hospital (*n*=59)	Tertiary hospital (*n*=716)	p - value
***Pseudomonas aeruginosa* isolates**	9 (1.2)	2 (3.4)	7 (1.0)	0.145
***Pneumonia klebsiella* isolates**	43 (5.5)	3 (5.1)	40 (5.6)	> 0.999
***Acinetobacter* isolates**	10 (1.3)	1 (1.7)	9 (1.3)	0.549
***Escherichia coli* isolates**	4 (0.5)	0 (0)	4 (0.6)	> 0.999
***Aerobacter cloacae* isolates**	2 (0.3)	0 (0)	2 (0.3)	> 0.999
***Bacillus proteus* isolates**	3 (0.4)	0 (0)	3 (0.4)	> 0.999
***Haemophilus influenzae* isolates**	10 (1.3)	1 (1.7)	9 (1.3)	0.549
***Pseudomonas maltophilia* isolates**	2 (0.3)	0 (0)	2 (0.3)	> 0.999
***Staphylococcus aureus* isolates**	1 (0.1)	0 (0)	1 (0.1)	> 0.999
***Streptococcus pneumoniae* isolates**	8 (1.0)	0 (0)	8 (1.1)	> 0.999

Notes: Values are presented as number (percentage).

Legend: AECOPD – acute exacerbation of chronic obstructive pulmonary disease.

## Discussion

Our results demonstrate that 86.2% of included AECOPD patients received antibiotic treatment during admission. The median antibiotic therapy duration was 9.0°days. Cephalosporins, penicillin, and quinolones were the most prescribed antibiotics. Furthermore, 56.5% of the included AECOPD patients underwent pathogen examinations. Importantly, a significantly lower proportion of patients receiving pathogen examinations was found in the second hospital group when comparing with the tertiary hospital group. Moreover, the most common bacterial pathogens isolated from the study population were *Pneumonia klebsiella*, *Acinetobacter*, *Haemophilus influenzae*, *Pseudomonas aeruginosa*, and *Streptococcus pneumoniae* isolates.

A high proportion (97.6%) of intravenous antibiotic use was observed in this study, and it might be partially understandable that the including AECOPD patients all needed to be admitted for treatment. And we are reasonable to speculate that the general situation of AECOPD patients in this study was unwell, and they could be categorized as severe AECOPD according to GOLD 2021 report. (Global, 2020) And this is consistent with the recommendation that intravenous antibiotic should be given to severely unwell AECOPD patients in NICE guideline. (NICE guideline [NG114], 2020) Of note, the definition of “severely unwell” in the guideline is lack of powerful supporting evidence and needs further investigations. While only 1% patients received both intravenous and oral antibiotics, which was significantly inconsistent with stepping-down mode for antibiotic use in NICE guideline. This aggressive choice of antibiotic administration may be associated with the unique health-care system in China. Health care resource distribution in China demonstrates inequalities, (Jin et al., 2015) medical resources in some poor areas are relatively limited. (Ma et al., 2016) Shortening length of hospital stay is beneficial to improving hospitals’ efficiency and controlling patients’ costs. (Xu et al., 2020) However, it is unacceptable that aggressive treatment is chosen with an aim of shortening length of hospital stay. Instead, physicians need to persevere in exploring the most suitable antibiotic treatment strategy for each AECOPD patient throughout the whole disease process. “Continuous and comprehensive reassessment” may be the core, and this is dependent on monitoring bacterial infection symptoms of phlegm and fever, bacterial infection-related laboratory indexes, and repeated etiological examination steadily and comprehensively.

The median antibiotic therapy duration from real-world data in this study was significantly longer than international guidelines, (Global, 2020; NICE guideline [NG114], 2020) which suggested a potential overuse of antibiotics in a portion of Chinese AECOPD patients. Furthermore, this study demonstrates a significant contrast between high levels of antibiotic use and low levels of bacterial pathogens isolated, which further suggests the likelihood of antibiotic overuse. Antimicrobial resistance (AMR) has become a global public health problem, and antibiotic overuse is a key driver of antimicrobial resistance. (Shallcross and Davies, 2014) AMR may lead to severe infections, complications, longer hospital stays and even increased mortality. (Llor and Bjerrum, 2014) In order to reduce the overuse of antibiotics in AECOPD, timely monitoring suspected bacteriological infections is of vital importance. Positive results from bacterial culture may provide powerful reference for rational antibiotic use. Herrera et al. (Herrera et al., 2016) reported that both swab and sputum specimens had good specificity in polymerase chain reaction (PCR) tests for the diagnosis of community-acquired pneumonia (CAP) caused by atypical bacteria, but there was a significantly lower sensitivity in swab specimens than in sputum specimens. Moreover, a recent French study showed that the diagnostic efficacy of pulmonary samples for Legionella pneumophila was clearly superior to that of nasopharyngeal aspirates in adult patients with CAP or AECOPD. (Robert et al., 2018) Furthermore, Cho et al. (Cho et al., 2012) found that sputum was more efficacious than nasopharyngeal swabs for the simultaneous detection of *Legionella pneumophila*, *Chlamydophila pneumoniae*, and *Mycoplasma pneumoniae* using multiplex PCR in CAP. Therefore, specimen type is crucial for accurately detecting bacterial infections, and the sensitivity of sputum specimens may be superior to that of swab specimens. This also suggests the necessity of improving the proportion of sputum specimens collected from AECOPD patients in China, especially in secondary hospitals. In our opinion, helpful ways to improve sputum collection and analysis are as follows: 1) Strengthening education for physicians-only physicians profoundly fully realize the importance of etiological evidence and the harmfulness of AMR, can the likelihood of patients receiving sputum collection and analysis be increased; 2) Enhancing the quality control of sputum tests-a qualified quality of specimen origin is the premise for accessing powerful etiological evidence, and monitoring the technical quality during pathogen culture process in different-grade hospitals is also of vital importance. Besides bacterial culture of clinical specimens, C-reactive protein (CRP) during admission is also a significant indicator for guiding rational antibiotic use in the treatment of AECOPD. (Clark et al., 2015; Butler et al., 2019; Prins et al., 2019) Butler et al. (Butler et al., 2019) reported that CRP-guided prescribing of antibiotics for AECOPD in primary care clinics led to a lower percentage of patient-reported antibiotic use and clinician-prescribed antibiotic use with no evidence of harm. Prins et al. (Prins et al., 2019) also found that use of CRP as a biomarker to guide antibiotic treatment in severe AECOPD patients resulted in a significantly reduced antibiotic treatment. Interestingly, it was found that bacterial detection rate was not associated with CRP level in an earlier study targeting AECOPD patients, (Clark et al., 2015) which suggested that further researches are still needed to confirm the guidance role of CRP in antibiotic use for AECOPD treatment. In this study population, CRP levels were not discussed due to inconsistency of CRP units among different involving study sites.

In this study, antibiotic use with a high coverage rate of *Pseudomonas aeruginosa* was observed, which was consistent with current international guidelines and national expert consensus. (Global, 2020; NICE guideline [NG114], 2020) Notably, we found that anti-*pseudomonas* cephalosporin and anti-*pseudomonas* penicillin were main choices for patients in tertiary hospitals; while anti-*pseudomonas* quinolones were more commonly prescribed in secondary hospitals, which revealed a concern of antibiotic use against World Health Organization (WHO) AWaRe list of antibiotics in Chinese hospitals. To better monitor the regional, national and global management of antimicrobial agents and reduce the development of AMR, WHO AWaRe list of antibiotics published in 2019 divided 180 common antibiotics into three classifications (access, watch and reserve). As a note, quinolones (including levofloxacin, moxifloxacin and ciprofloxacin etc.) were categorized into “watch” antibiotics, which had higher bacterial resistance potential and need to be prioritized as key targets of stewardship programs and monitoring. (World Health Organization, 2019) Besides, the European Medicines Agency’s Pharmacovigilance Risk Assessment Committee (October 2018) has also recommended restricting the use of quinolones following a review of disabling and potentially long-lasting side effects including muscle, tendon, bone and the nervous system damage. This puts forward new requirements for better preventing antibiotic overuse in Chinese hospitals, especially in primary care. A recent study involving 625 physicians from 67 primary care facilities in China demonstrated that physicians had limited knowledge about antibiotic prescriptions with an average 54.55% correct answers to 11 questions. (Liu et al., 2019) And a systematic review has reported that education may be a helpful intervention to increase physicians’ knowledge level on antibiotic use and to improve antibiotic prescribing behavior. (Roque et al., 2014) Thus, targeted education programs of antibiotic use for physicians may be needed to broaden in different-grade Chinese hospitals, which is meaningful to rational antibiotic use in AECOPD management.

Regarding the types of cultured bacterial pathogens, *Pneumonia klebsiella* isolates, *Acinetobacter* isolates, *Haemophilus influenzae* isolates, *Pseudomonas aeruginosa* isolates, and *Streptococcus pneumoniae* isolates were commonly detected, which was consistent with the findings of previous studies. Millares et al. (Millares et al., 2014) reported that *Streptococcus*, *Pseudomonas, Moraxella, Haemophilus, Neisseria, Achromobacter* and *Corynebacterium genera* were found by an increase in the relative abundance over 20 % during exacerbations of COPD. In a Chinese study, the predominant bacteria included *Pseudomonas aeruginosa, Klebsiella pneumoniae, Haemophilus influenzae* and *Streptococcus pneumoniae*. (Ye et al., 2013) In this study, only 91 AECOPD patients had more than one bacterial culture result during admission, and 29 (31.9%) out of 91 AECOPD patients stopped antibiotics when bacterial culture results came back negative or adjusted antibiotics when new isolated pathogens appeared. Precision medicine has drawn more and more attention recently, and individualized treatment is also necessary for AECOPD patients. In order to dynamically adjust the most suitable antibiotic treatment for AECOPD patients, it is necessary for physicians to reexamine potential pathogens timelier and prescribe antibiotics based on etiological evidence throughout the admission.

This study had some limitations. Firstly, the prescribing of antibiotics according to the international nonproprietary names (INNs) were not listed in this study, which might cause the absence of a more detailed pharmacotherapeutic approach. Secondly, there were missing data (e.g., duration of antibiotic use) in a portion of the patients. Thirdly, due to the limited follow-up duration, no prognostic analysis was performed to investigate the relationship between antibiotic use and long-term clinical outcomes.

## Conclusion

To the best of our knowledge, this is the first multicenter retrospective observational study to describe the real-world antibiotic use in AECOPD patients in Chinese hospitals of different grades. This study demonstrates that antibiotics are extensively prescribed for AECOPD patients in China. Notably, an antibiotic overuse may exist in the treatment of AECOPD in China, and measures should be taken to prevent the overuse of antibiotics and potential AMR in Chinese AECOPD patients.

## Data Availability

The datasets presented in this article are not readily available because “The raw data supporting the conclusions of this article will be made available with reasonable request.” Requests to access the datasets should be directed to “chenyan99727@csu.edu.cn”.
